# A genome-wide analysis of putative functional and exonic variation associated with extremely high intelligence

**DOI:** 10.1038/mp.2015.108

**Published:** 2015-08-04

**Authors:** S L Spain, I Pedroso, N Kadeva, M B Miller, W G Iacono, M McGue, E Stergiakouli, G D Smith, M Putallaz, D Lubinski, E L Meaburn, R Plomin, M A Simpson

**Affiliations:** 1 Department of Medical and Molecular Genetics, Division of Genetics and Molecular Medicine, King's College London, London, UK; 2 Department of Psychology, University of Minnesota, Minneapolis, MN, USA; 3 MRC Integrative Epidemiology Unit, University of Bristol, Bristol, UK; 4 Duke University Talent Identification Program, Duke University, Durham, NC, USA; 5 Department of Psychology and Human Development, Vanderbilt University, Nashville, TN, USA; 6 Department of Psychological Sciences, Birkbeck, University of London, London, UK; 7 MRC Social, Genetic and Developmental Psychiatry Centre, Institute of Psychiatry, Psychology and Neuroscience, King's College London, London, UK

## Abstract

Although individual differences in intelligence (general cognitive ability) are highly heritable, molecular genetic analyses to date have had limited success in identifying specific loci responsible for its heritability. This study is the first to investigate exome variation in individuals of extremely high intelligence. Under the quantitative genetic model, sampling from the high extreme of the distribution should provide increased power to detect associations. We therefore performed a case–control association analysis with 1409 individuals drawn from the top 0.0003 (IQ >170) of the population distribution of intelligence and 3253 unselected population-based controls. Our analysis focused on putative functional exonic variants assayed on the Illumina HumanExome BeadChip. We did not observe any individual protein-altering variants that are reproducibly associated with extremely high intelligence and within the entire distribution of intelligence. Moreover, no significant associations were found for multiple rare alleles within individual genes. However, analyses using genome-wide similarity between unrelated individuals (genome-wide complex trait analysis) indicate that the genotyped functional protein-altering variation yields a heritability estimate of 17.4% (s.e. 1.7%) based on a liability model. In addition, investigation of nominally significant associations revealed fewer rare alleles associated with extremely high intelligence than would be expected under the null hypothesis. This observation is consistent with the hypothesis that rare functional alleles are more frequently detrimental than beneficial to intelligence.

## Introduction

General cognitive ability, usually called *intelligence*, indexes the covariance among diverse cognitive tests and is one of the best predictors of important life outcomes such as education, occupation, and mental and physical health and illness.^1, 2, 3^ Extensive quantitative genetic research consistently indicates that about half of the total variance of intelligence can be accounted for by genetic factors.^4, 5^

Unlike psychiatric disorders, intelligence is a normally distributed quantitative trait with a positive end of high performance as well as a problematic end of intellectual disability.^6^ Unlike other normally distributed behavioural traits, such as personality that assess average behaviour across situations, intelligence indexes maximal performance on a battery of tests, analogous to musical ability and athletic ability.^7^ These features of intelligence make it especially well suited phenotypically as a target for *positive genetics*—that is, considering the positive end of the normal distribution of genetic effects rather than focusing on the negative effects of genetic mutations.^8^ The investigation of high intelligence could be of particular utility for finding DNA variants responsible for the heritability of the entire distribution of intelligence. The quantitative genetics model proposes that quantitative traits are influenced by multiple genes distributed as a bell-shaped curve, which implies that extremely high intelligence can be achieved only if an individual has many of the positive alleles and few of the negative alleles that affect intelligence. In other words, a large group of individuals of extremely high intelligence should be enriched for alleles associated with intelligence and thus yield increased power to detect associations.^9^

This quantitative genetics model could be construed to suggest that the extreme low end of the distribution of intelligence is the mirror image of the extreme high end. However, it seems likely that new mutations can more easily disrupt than improve a finely tuned performance system, which implies that genetic variation responsible for the heritability of the entire distribution of intelligence may be more likely to be found for extremely high rather than extremely low intelligence. Strong empirical support for this hypothesis comes from recent studies based on 360 000 sibling pairs and 9000 twin pairs from 3 million 18-year-old males with cognitive assessments administered as part of conscription to military service in Sweden. These studies concluded that extremely high intelligence is as familial and heritable as the rest of the distribution^10^ but that extremely low intelligence—severe intellectual disability—is not familial or heritable.^11^ The latter finding, predicted initially in 1938 by Lionel Penrose,^12^ suggests that severe intellectual disability is aetiologically distinct from the normal distribution of intelligence, which may explain why DNA variants recently found to be associated with severe intellectual disability appear to be rare non-inherited *de novo* mutations.^13, 14, 15^

Research to date has had limited success in identifying DNA variants responsible for the heritability of intelligence;^16, 17, 18, 19, 20, 21^ the ‘missing heritability' problem, is not unique to intelligence—it is pandemic throughout the life sciences.^22^ What has been learned from the whirlwind of genome-wide association (GWA) studies during the past few years is that the biggest effect sizes for associations between DNA variants and traits—common disorders as well as quantitative dimensions—are much smaller than expected.^23^ For example, in a GWA meta-analysis of intelligence for nearly 18 000 children, the largest effect size accounts for <0.2% of the variance.^16^ Similar effect sizes were reported in a recent GWA meta-analysis of intelligence for nearly 54 000 adults.^21^ If the largest effects are so small, the smallest effects will be infinitesimal, which means that they will be difficult to detect in GWA studies and even harder to replicate.^24^

One strategy for addressing this ‘missing heritability' problem is a brute-force approach in which larger samples are amassed in order to detect associations of smaller effect size using the common single-nucleotide polymorphisms (SNPs) available on genome-wide DNA arrays.^25^ An alternative strategy is to consider the role of low frequency and rare alleles. The role of rare variants is well established in monogenic syndromes for which lowered intelligence is a symptom.^26, 27, 28^ However, it remains unclear to what extent low frequency and rare alleles contribute to the genetic architecture of complex traits like intelligence in the general population, although there have been reports finding no association between intelligence and low frequency copy number variants^29, 30, 31^ and between intelligence and rarer exonic variants;^32^ one pilot study reported more minor (<1% minor allele frequency (MAF)) exonic alleles in a higher-IQ group.^33^

This study capitalises on the increased power of association at the high extreme of intelligence as a strategy to expedite the discovery of alleles contributing to variation in intelligence throughout the distribution. Another novel feature of this study is its focus on low frequency protein-coding variants in exons rather than genome-wide common variants. The combination of these two features is expected to be synergistic because the gain in power that comes from studying the high extreme is particularly pronounced for low frequency and rare alleles.^9, 34^

For these reasons, we undertook a threshold-selected case–control study by obtaining DNA from individuals representing approximately the top 0.03% of the intelligence distribution and comparing them with controls drawn from an unselected population cohort. Within this extreme sampling design, we investigate the role of common and low frequency protein-coding variation in intelligence.

## Materials and methods

### Samples

Data from four samples were used in this study: a sample of high-intelligence cases, a control sample and two representative samples used to extend our case–control results to individual differences in the population. The project received ethical approval from the King's College London Research Ethics Committee (reference number PNM/11/12-51) and from the European Research Council Executive Agency (reference number Ares(2012)56321).

#### High-intelligence cases (HiQ)

Individuals were recruited from the Duke University Talent Identification Program (TIP), a non-profit organisation established in 1980 and dedicated to identifying and fostering the development of academically gifted children^35^ (see http://www.tip.duke.edu). Individuals were selected from the United States for participation in the HiQ study on the basis of performance on the Scholastic Assessment Test (SAT) or American College Test (ACT) taken at age 12 rather than the usual age of 18 years. A composite that aggregates verbal and mathematics SAT and ACT scores correlates >0.80 with intelligence tests and it is estimated that the TIP program recruits from the top 3% of the intelligence distribution.^36^ For this study (HiQ), cases were selected from the top 1% of these TIP individuals, representing approximately the top 0.03% of the intelligence distribution. Exome array genotyping data were available for 1759 HiQ individuals who reported their ethnicity as ‘white'. Following genotyping and quality control (see below), the HiQ sample included 1409 individuals (818 males).

#### Unselected controls (MTFS)

The Minnesota Twin Family Study (MTFS) is a population-based study of twins and their parents recruited from population databases for twins born between 1972 and 1994 in the state of Minnesota in the United States and tested at ages 11 or 17 (refs 37, 38) (see https://mctfr.psych.umn.edu/). Cognitive ability was measured using a short form of the Wechsler Adult Intelligence Scale-Revised (WAIS-R) for participants age 16 or older, or the Wechsler Intelligence Scale for Children-Revised (WISC-R) for those younger than 16. The short form consisted of two verbal subtests (information and vocabulary) and two performance subtests (block design and picture arrangement). An estimate of full-scale IQ was determined by prorating the scaled scores for these four subtests; the effective ceiling for the IQ scores was about 150. We excluded individuals with IQ <70 and those who were not white. We included 3253 unrelated individuals (1500 males) from MTFS for whom exome array genotyping and intelligence data were available.

#### Extension sample 1

The Twins Early Development Study (TEDS) is a longitudinal UK-based population sample of over 15 000 families with twins born in England and Wales between 1994, 1995 and 1996 and identified from birth records^39^ (see http://www.teds.ac.uk). Individuals were tested at 12 years using two verbal and two nonverbal measures. Test scores were adjusted for age within each testing period, and first principal component scores were derived using principal component analysis implemented in R. The TEDS sample with genotyping and age 12 intelligence data included 4072 unrelated individuals (1780=males). Individuals with severe recurrent medical problems or severe perinatal medical problems were excluded, as were individuals whose first language was not English. Only included individuals who reported their ethnicity as ‘white' were included.

#### Extension sample 2

The Avon Longitudinal Study of Parents and Children (ALSPAC) is a prospective birth cohort, which recruited pregnant women with expected delivery dates between April 1991 and December 1992 from Bristol, UK. A total of 14 541 pregnant women were initially enrolled with 14 062 children born. Detailed information on health and development of children and their parents were collected from regular clinic visits and completion of questionnaires. A detailed description of the cohort has been published previously.^40, 41^ The study website contains details of all the data that is available through a fully searchable data dictionary: http://www.bris.ac.uk/alspac/researchers/data-access/data-dictionary/. Ethical approval was obtained from the ALSPAC Law and Ethics Committee and the local ethics committees. Intelligence in ALSPAC was measured with a short version of the Wechsler Intelligence Scale for Children (WISC-III) at 8 years of age. Verbal (information, similarities, arithmetic, vocabulary and comprehension) and performance (picture completion, coding, picture arrangement, block design and object assembly) subscales were administered, each subtest was age scaled according to population norms and a summary score for total IQ derived. The sample we used included 6461 unrelated individuals (3209 males) who had been tested for intelligence using a short form of the WISC-III assessment at age 8 years (mean=8.7 years, s.d.=0.30) and whose reported ethnicity was ‘white'.

### Genotyping and quality control procedures

DNA from cheek swabs was collected by post from 1759 HiQ individuals and extracted in accordance with standard protocols. DNA was genotyped using the Illumina HumanExome-12v1-1 array according to the manufacturer's protocols. The control cohort were previously genotyped as part of the MTFS using the Illumina HumanExome-12v1_A array (San Diego, CA, USA). We limited our analyses to 242 901 variants present on both versions of the array.

Provisional genotypes were called using Illumina's GenomeStudio v2011.1 software. Sample QC was performed on the HiQ and MTFS cohorts separately using PLINK and R. Samples were removed from subsequent analyses on the basis of call rate (<0.95), suspected non-European ancestry, heterozygosity (±4 s.d. from the mean), array signal intensity (>4 s.d. from the mean) and relatedness (Supplementary Table 1). SNPs were removed based on the criteria of call rate (<0.99), deviations from Hardy–Weinberg equilibrium (*P* <1 × 10^−4^) and GenomeStudio cluster separation score (<0.4). Duplicate assays, tri-allelic variants and insertion/deletions were also removed from downstream analysis (Supplementary Table 2). The ZCALL program (see Web resources section) was used to augment the genotype calling for samples and SNPs that passed the initial QC. Only samples that achieved a call rate >99% after ZCALL genotype refinement were included in the analysis.

After a preliminary association analysis, we manually reviewed the cluster plots for 10 000 variants (ordered by association *P*-value) using the EVOKER tool (see Web resources section) and recalled genotypes or removed SNPs where necessary. During this process, 1083 SNPs were removed from the analysis owing to poor clustering. A total of 227 858 variants passed QC and were available for analysis (Supplementary Table 2). The genotype data were converted to VCF format for downstream analysis using tools within the EPACTS framework (see Web resources section).

#### *Genotyping in the TEDS and ALSPAC extension samples*

Genotyping of rs4713668 was undertaken in the TEDS and ALSPAC samples with a TaqMan SNP genotyping assay (Applied Biosystems, Life Technologies Ltd, Paisley, UK).

### Statistical analysis

#### Discovery study

Single-SNP analysis: Analysis was restricted to the 199 039 variants on the HumanExome array that were successfully genotyped and predicted to be functional (non-synonymous, stop gain/loss, start gain/loss or splice site altering) based on annotation performed in EPACTS v3.2.3 using the ‘anno' option and the default ‘refFlat' file (refFlat_hg19.txt.gz) (Supplementary Table 3). A total of 114 416 variants were polymorphic (minor allele count >0) in the complete data set of HiQ and MTFS individuals, of which 70 112 were polymorphic in each of the HiQ and MTFS data sets. In all, 17 309 variants (MAF >0.01) on the array were assessed for association with a linear mixed model using the EPACTS v3.2.3 implementation of EMMAX. Population stratification was corrected for using a kinship matrix, which was created with the ‘make-kin' function in EPACTS using autosomal SNPs with a call rate of >0.99 and MAF >0.01 in both HiQ cases and MTFS controls.

#### Replication of previously reported cognition-associated variants

Seventeen cognition-related GWA studies were identified in the NHGRI GWAS Catalogue (as of March 2014, Supplementary Table 4), which reported 567 SNPs with *P* <1 × 10^−4^. We identified variants on the exome array that were either in linkage disequilibrium (LD; *r*^2^ >0.6) or within 250 kb of the previously published variant (Supplementary Table 5). Pairwise LD relationships were calculated from 1000 genomes (version 3.20101123) EUR haplotypes using a 500 kb window in the vcftools program (see Web resources section).

#### Extension studies

In order to further investigate suggestive hits (*P* <5 × 10^−5^) to emerge from the main case–control single-SNP analysis, we performed a quantitative trait analysis using the full distribution of IQ scores. In the MTFS data set, a kinship matrix was used to correct for population structure and association was assessed using a linear mixed model implemented in EMMAX. In the TEDS and ALSPAC data sets, allelic tests of association were performed using linear regression in PLINK.

#### Gene-based rare variant analysis

A total of 104 644 rare exonic variants (MAF <0.01; see Supplementary Table 3) were tested for association in the discovery case–control sample using a variant-collapsing method implemented within EPACTS v3.2.3. The analysis was performed using two different methods, a burden analysis (emmaxCMC) and the optimal sequence kernel association test (SKAT-O) method (see Web resources section). Both methods, as implemented in EPACTS, use EMMAX to correct for population stratification. Variants within each gene were assigned to groups by function to form three different combinations of variants to be analysed separately: (1) StopGain, Essential Splice Site (ESS) and NonSynonymous (NS), (2) StopGain, ESS and predicted damaging by polyphen2 (PP2) and (3) StopGain and ESS.

#### Estimation of phenotypic variance explained by genotype data

Kinship matrices were calculated with LDAK software^42^ using all variants and estimating weights for all SNPs in a chromosome simultaneously. Principal components, used as covariates for heritability estimation, were calculated using the GCTA software^43^ for variants with MAF >0.05 and polymorphic in both cases and control data sets. We used GCTA to fit a linear mixed model to estimate the fraction of phenotypic variance explained by genotyped variants on the exome array, on both the observed and liability scale. For individual SNP association, we transformed the case–control effect size into the corresponding variance explained on a liability scale.^44^

#### Increaser/decreaser ratio

The ratio of the number of associated variants for which the minor allele is at elevated frequency in the high-intelligence cohort to those in which the minor allele is at elevated frequency in the control cohort (increaser/decreaser (I/D) ratio) was calculated based on the method reported recently by Chan *et al.*^45^ The beta values for each SNP's association were recalculated to reflect the effect of the minor allele. The I/D ratio was calculated for different *P*-value thresholds (0.01 and 0.001) and MAF ranges (0.00–0.01, 0.01–0.05, 0.05–0.15, and 0.15–0.50). SNPs were grouped using the LD-based clumping procedure in PLINKv1.07 (—clump); SNPs that had an *r*^2^ >0.1 with a SNP with stronger evidence of association in the locus (defined as a 1 MB window) were removed. In all, 1000 permutations of the single-variant association analysis were performed to determine the expected distribution under the null hypothesis and to allow the calculation of *P*-values for significance. The permuted phenotypes were generated using ‘make-perm-pheno' function in PLINK, which preserves the case–control sample number. LD pruning and the generation of I/D allele counts and ratios were repeated for each permutation as described above.


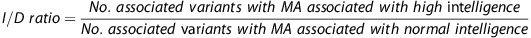



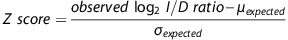


where μ is the mean and σ is the s.d. of the expected log_2_ I/D ratios. Squared *Z*-scores were converted to two-tailed *P*-values in R using the chi-squared distribution with 1 degree of freedom.

## Results

### Study design and statistical power

**Figure 1:**
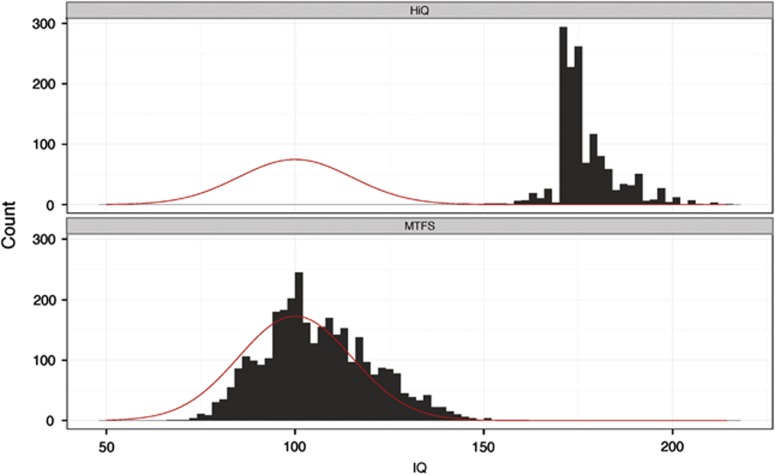
Extreme sampling of the high cognitive ability cohort. Distribution of general cognitive ability, measured on a standardised IQ scale (mean 100, s.d. 15), of the high cognitive ability cohort (HiQ, *n*=1409, top panel) and the control cohort (MTFS, *n*=3253, lower panel). The red lines illustrate the expected distribution of IQ scores with unbiased sampling.

Following genotyping and quality control, the HiQ sample included 1409 individuals selectively sampled from the upper tail of the intelligence distribution and 3253 unselected MTFS controls. A previous study of intelligence in the TIP cohort derived a conversion scale relating SAT scores at age 12 years to the familiar IQ scale (normal distribution with a mean of 100 and s.d. of 15).^36^ Using this conversion factor, the successfully genotyped HiQ cohort has a mean estimated IQ of 176, ranging from 144 to 214 with 93.6 % of the cohort with estimated IQ scores >170 (Figure 1). This distribution of estimated IQ scores illustrates the extreme sampling of individuals from the high end of the trait distribution in comparison with the unselected MTFS controls (Figure 1). A threshold-selected case–control analysis of this design provides 80% power to detect associated variants explaining >0.0015 of the trait variance assuming an additive model and *α*=1 × 10^−7^.

### Single-variant analysis

A case–control association analysis was undertaken with high quality genotypes at 70 112 common (>5%) and low frequency (1–5%) protein-altering and splice site SNPs from 1409 individuals from the HiQ cohort and 3253 control subjects. The quantile–quantile plot indicated adequate control for confounders (λ_GC_=1.06, Supplementary Figure 1). We explored whether any loci previously reported to be associated with cognition-related traits showed evidence of association in our data set. For the 567 SNPs that had been previously reported, seven were within 250 kb of a SNP that showed evidence of association (*P*_CC_ <0.01) with intelligence in our data set (Supplementary Table 5). One SNP rs4713668 (*P*=4.62 × 10^−4^) identified in our study, was in LD (*r*^2^=0.65) with rs3227, which is one of the three variants recently reported with genome-wide significant evidence of association with educational attainment.^46^

Our single-SNP analysis of 70 112 protein-altering and splice site SNPs that were polymorphic in both the HiQ and MTFS data sets did not reveal any associations that satisfied the genome-wide significant threshold (Supplementary Table 6). However, the most strongly associated SNP was a missense variant located in *PLXNB2* at 22q13 (rs28379706, c.A952G, p.K318E; *P*_CC_=1.31 × 10-5, beta=−0.04±0.009, odds ratio=0.76 (0.69–0.83)), which explains an estimated 0.16% of the variance (Supplementary Table 7). Given the unique sampling design used in the HiQ sample, an equivalent case–control replication cohort is not currently available. However, the quantitative genetic model suggests that variants associated with high intelligence will also associate with individual differences in intelligence throughout the distribution. We therefore sought to evaluate the role of rs28379706 in the normal distribution of intelligence in three independent unselected population cohorts. A nominally significant association with intelligence was observed in the MTFS cohort (*P*_QT_=0.02). However, we did not observe a significant association in either the TEDS or ALSPAC cohorts (*P*_QT_=0.56 and *P*_QT_=0.94, respectively). The sample sizes of these cohorts, together with the small effect size of rs28379706 observed in the case–control analysis, indicates that our power to detect the association is limited in these cohorts. For this reason, it warrants mention that the allelic effect was directionally consistent in the discovery case–control study and all three population cohorts (Supplementary Table 7).

### Gene-based rare variant analysis

To address the hypothesis that burden of rare variants (MAF <0.01) with genes is associated with intelligence, we grouped variants within each gene by function and applied two gene-based analyses, CMC and SKAT-O (see Materials and methods section). A limitation of the CMC method is its reduced power to detect association when alleles that increase and decrease intelligence are present in the same gene; SKAT-O models protective and risk effects. Both tests were performed using a linear mixed model to control for confounders (quantile–quantile plots in Supplementary Figure 2). No individual gene reached our study-wide significance threshold using either test (*P* <3.18 × 10^−6^, based on the analysis of 15 686 genes, Supplementary Table 8). *PLXNB2,* which harboured the most strongly associated common variant, did not demonstrate compelling evidence of association of rare variation in the gene-based analysis (StopGain_ESS_NS CMC *P*=0.0036; SKAT-O *P*=0.0042).

### Variance explained by protein-altering variants

To establish the contribution of the genotyped exonic SNPs to the heritability of intelligence, we used a linear mixed model implemented in the genome-wide complex trait analysis (GCTA) framework to estimate the variance explained by the 70 112 functional SNPs used in this analysis. We converted the variance to a liability scale assuming a population prevalence of 0.0003 based on the ascertainment criteria for the HiQ sample. The results suggest that the genotyped protein-altering SNPs (or non-coding variants that they are tagging) explain 17.4% (1.7% s.e.) of the variance in intelligence on the liability scale. The observation that a substantial proportion of the variance of liability to high intelligence could be explained by these SNPs suggests that these data could be further explored to gain insight into the genetic architecture of intelligence.

### Ratio of minor allele increaser to decreaser associations

A recent study of the genetic architecture of complex human diseases and traits elegantly illustrated that an elevated ratio of the direction of allelic effect (risk to protective in a typical disease case–control study) of nominally associated rare alleles can be interpreted as a signature of polygenic inheritance from lower frequency variants.^45^ The ratio of associated risk to protective variants is substantially influenced by unequal sizes of the case and control cohorts or uneven population stratification. The ratio must therefore be evaluated in the context of the null distribution of ratios that is generated through permutation. A risk to protective ratio for rare alleles that is high in comparison with the null may be attributed to negative selection keeping detrimental rare alleles at low frequency. High intelligence may be considered as an advantageous rather than detrimental trait. As a result, we may expect to observe a reduction of the ratio of rare IQ-increasing alleles to IQ-decreasing alleles (the I/D ratio), indicating an enrichment of rare intelligence-decreasing alleles or depletion of rare intelligence-increasing alleles. To test this supposition, we evaluated this I/D ratio in our data set (see Materials and methods section, Table 1). We observe a significantly lower I/D ratio for variants within the lowest frequency class (MAF <0.01) evaluated in comparison with the null. The low ratios are observed for both 73 variants with a *P*_CC_<0.001 (I/D_obs_=8.000, I/D_exp_=32.159, *Z*-score=−2.67, *P*_I/D_=0.001) and also 467 variants with *P*_CC_ <0.01 (I/D_obs_=3.396, I/D_exp_=6.400, *Z*-score=−3.28, *P*_I/D_=0.008). These results indicate that fewer rare alleles are associated with high intelligence or more rare alleles are associated with lower intelligence than would be expected.

## Discussion

We have described the results from the first investigation of exome variation in individuals of extremely high intelligence. The sampling design from the extreme tail of the distribution provides increased power for the detection of alleles associated with high intelligence. However, this unique sample also presents challenges including the absence of a direct replication sample. The strongest associated single variant identified in the discovery case–control analysis is a non-synonymous variant located in the *PLXNB2* gene, whose protein product has been previously implicated in neuronal migration. Given the unavailability of a direct replication sample, we sought to evaluate the variant's association with individual differences in intelligence across the normal distribution, aware that power would be limited because the SNP accounted for only 0.16% of variance of a liability model comparing the extremely high intelligence group and unselected controls. Using this approach, we were unable to replicate this association observed in the case–control study, although it is noteworthy that the same direction of allelic effect was observed in all three extension cohorts together with the case–control study.

Evaluating the role of all of the genotyped protein-coding variation on individual differences in intelligence strongly suggests that such variation does contribute a substantial proportion (about one-fifth) to the variance of the trait. The failure to detect any single robust association suggests that the contribution emanates from multiple alleles with effect sizes smaller than this study's ability to detect robustly. It is also important to note that the variance explained by this group of functional SNPs could also be a consequence of linkage disequilibrium between the coding variants and non-coding true ‘causal' SNPs and is therefore an upper bound of the variance explained by this group of coding variants. It should also be noted that the heritability of individual differences in intelligence increases substantially throughout the lifespan, from about 20% in early childhood, 40% in adolescence and 60% or higher in adulthood.^47^ As a result the samples used in this study were tested in adolescence, our estimates of heritability may not be as high as they would be in a sample of older adults.

The observed ratio of I/D rare alleles nominally associated with high intelligence indicates that we observe fewer rare alleles that are associated with high intelligence or more rare alleles that are associated with normal intelligence than would be expected. Although we cannot exclude the possibility that this signal is driven by asymmetric population stratification, this finding is consistent with the hypothesis that rare alleles are more frequently detrimental to intelligence. This hypothesis is important when considering appropriate study designs for future investigations. We have demonstrated here the increased power from extreme sampling as compared with unbiased sampling designs. For example, a study that maximises power to detect rare alleles associated with decreased intelligence would ideally seem to use a low-intelligence sample selected to be as extreme as the high-intelligence sample in order to ensure balanced sampling. However, this design may not be advisable because, as noted earlier, extremely low intelligence is not familial or heritable.^11^ Instead, a design seems warranted that compares the high-intelligence group with a group representative of the low end of the heritable normal distribution of intelligence (that is, between 1 and 2 s.d. below the mean).

As mentioned in the Introduction section, one approach to the ‘missing heritability' problem is a brute-force approach that moves toward ever-larger consortia in order to increase power. Although an intelligence GWA consortium of 54 000 adults was recently reported, a polygenic score based on this GWA only accounted for about 1% of the variance of intelligence scores in independent samples,^21^ which suggests that very much larger samples will be needed to narrow the missing heritability gap. This study is novel in suggesting two alternative strategies to increase power. One strategy is specific to intelligence and other normally distributed traits: selecting individuals extremely high in intelligence. The other strategy is to move beyond the common SNPs that have been the focus so far of GWA studies to consider functional exonic SNPs and rarer SNPs. Although combining these two novel strategies has suggested that some of the missing heritability could reside in functional and rarer SNPs, our results indicate that these strategies are not a panacea to the problem of missing heritability. Being optimists, we are hopeful that new strategies will be developed, especially as whole-genome sequencing comes on line, which will complement the common-SNP, exonic-SNP and extreme sampling strategies that have been tried so far.

Some remarks about the phenotype under analysis are in order because they point to a direction for future research as well as a limitation. Individuals in the profoundly gifted (IQ >170) group were identified by above-level assessment procedures, which involved administering standardised college-entrance measures of quantitative and verbal reasoning to intellectually talented young adolescents. A composite of quantitative and verbal reasoning was used to capture general intelligence, which is justified for genetic and neurocognitive reasons.^6^ Such a composite based on above-level assessment has been shown to capture extremely high levels of general intellectual functioning with precision, well beyond 4 s.d. above the population mean.^36, 48^ However, quantitative reasoning and verbal reasoning predict different outcomes. For example, 25-year longitudinal studies have revealed that differences in the quantitative versus verbal prowess of profoundly gifted youth portend contrasting occupational and creative accomplishments in scientific/technical versus humanistic/linguistic endeavours.^49, 50^ For this reason, future research might profit by considering quantitative and verbal reasoning separately. In addition, a specific cognitive ability missing from this study, spatial ability, should be added to the mix because spatial ability predicts occupational and creative accomplishments beyond quantitative and verbal reasoning abilities.^51, 52^

This investigation of protein-altering variation in high intelligence provides clear insight in the role of this class of genetic variation in the architecture of the trait. A common theme emerging from genetic studies of intelligence, similar to all complex traits and common disorders, is its highly polygenic nature with its heritability explained by many variants of small effect. Although the unique extreme sampling design used in this study provides improved power to detect associations in certain situations it has also provided challenges for direct replication. Nevertheless, the evidence for the contribution of protein-altering variants to the heritability of intelligence and the evidence that rare functional alleles are detrimental to intelligence provides a framework for defining the role of individual rare alleles. However, we did not find any individual protein-altering variants that are reproducibly associated with extremely high intelligence. Thus, despite the power of sampling from the extreme high end of the distribution of intelligence, we conclude that these results primarily highlight the complex genetic architecture of intelligence.

## Figures and Tables

**Table 1:** Evaluation of the I/D ratio across four MAF ranges (0–0.01, 0.01–0.05, 0.05–0.15 and 0.15–0.50) and two thresholds of nominal significance in the case–control analysis (*P*_CC_ <0.01 and *P*_CC_ <0.001)

*MAF range*	*Assoc.* P	*No. SNPs*	*Obs. I/D ratio*	*Exp. I/D ratio*	*Observed log2(I/D)*	*Expected log2(I/D) (s.d.)*	Z*-score*	P_*I/D*_
0–0.01	0.01	467	3.396	6.400	1.764	2.652 (0.271)	−3.282	0.001
0–0.01	0.001	73	8.000	32.159	3.000	4.861 (0.697)	−2.668	0.008
0.01–0.05	0.01	62	2.211	1.435	1.144	0.473 (0.369)	1.819	0.069
0.01– 0.05	0.001	6	6.000	2.818	2.585	1.035 (1.222)	1.268	0.205
0.05–0.15	0.01	31	1.000	1.231	0.000	0.209 (0.510)	−0.410	0.682
0.05–0.15	0.001	2	0.500	1.703	−1.000	0.307 (1.211)	−1.079	0.280
0.15–0.50	0.01	60	0.903	1.098	−0.147	0.075 (0.416)	−0.533	0.594
0.15–0.50	0.001	6	0.667	1.552	−0.585	0.103 (1.264)	−0.544	0.586

Abbreviations: I/D, increaser/decreaser; MAF, minor allele frequency; SNP, single-nucleotide polymorphism. The expected I/D ratios were calculated using 1000 permutations of the phenotype to determine the null distribution. The chi-square *P*_I/D_ values assess the difference between the observed and expected I/D ratios.
