# Supplementary material for: A genome-wide analysis of putative functional and exonic variation associated with extremely high intelligence
Source: Mol Psychiatry. 2015 Aug 4;21(8):1145–51. doi: 10.1038/mp.2015.108 (PMC4650257; doi:10.1038/mp.2015.108)
Supplement: Supplementary Information [file mp2015108x1.doc]

**Supplementary Information**

**A genome-wide analysis of putative functional and exonic variation associated with extremely high intelligence**

Sarah L. Spain PhD^1✝^, Inti Pedroso PhD^1✝^, Neli Kadeva MSc^1^, Mike B. Miller PhD ^2^, William G. Iacono PhD^2^, Matt McGue PhD^2^, Evie Stergiakouli PhD^3^, George Davey Smith PhD^3^, Martha Putallaz PhD^4^, David Lubinski Phd^5^, Emma L. Meaburn PhD^6^, Robert Plomin PhD^7*^, Michael A. Simpson PhD^1*^

^1^ Division of Genetics and Molecular Medicine, King’s College London, London, UK, SE1 9RT

^2^ Department of Psychology, University of Minnesota, Minneapolis, MN 55455, USA

^3^ MRC Integrative Epidemiology Unit, University of Bristol, Bristol BS8 2BN, UK

^4^ Duke University Talent Identification Program, Duke University, Durham, NC 27701, USA

^5^ Department of Psychology and Human Development, Vanderbilt University, Nashville, TN 37203, USA

^6^ Department of Psychological Sciences, Birkbeck, University of London, London, WC1E 7HX, UK

^7^ MRC Social, Genetic and Developmental Psychiatry Centre, Institute of Psychiatry, Psychology & Neuroscience, King's College London, London SE5 8AF, UK

^✝^ These authors contributed equally

*Correspondence to:

Dr Michael Simpson

Division of Genetics and Molecular Medicine

Guy’s Hospital

Great Maze Pond

London

SE1 9RT

Email: michael.simpson@kcl.ac.uk

Prof Robert Plomin

MRC SGDP Centre

Institute of Psychiatry, Psychology & Neuroscience

DeCrespigny Park

Denmark Hill
London

SE5 8AF

Email: robert.plomin@kcl.ac.uk

**Supplementary Figure 1:** Quantile-quantile plot of the p-values from the discovery case-control association analysis of single SNPs.


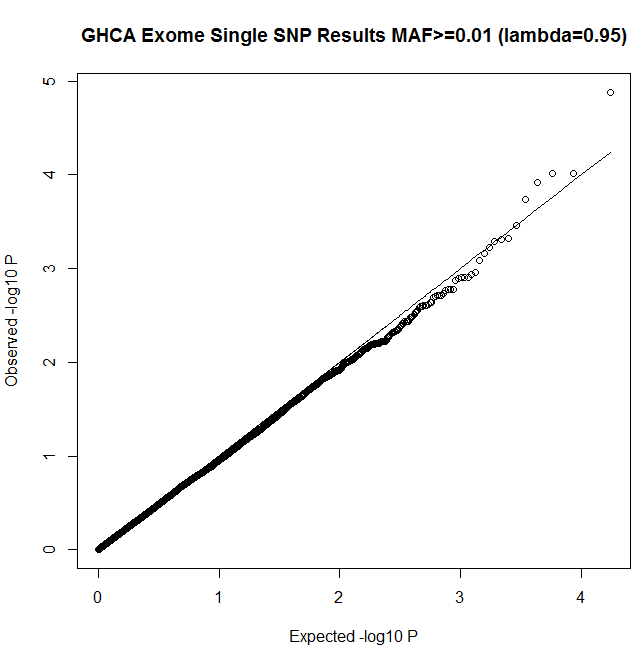


**Supplementary Figure 2:** QQ plots for the gene-based analysis for the three combinations of groups of SNPs (StopGain (SG), Essential Splice Site (ESS), nonsynonymous (NS) and predicted damaging in polyphen2 (PP2) using the SKAT-O method (top panel) and the CMC method (lower panel).


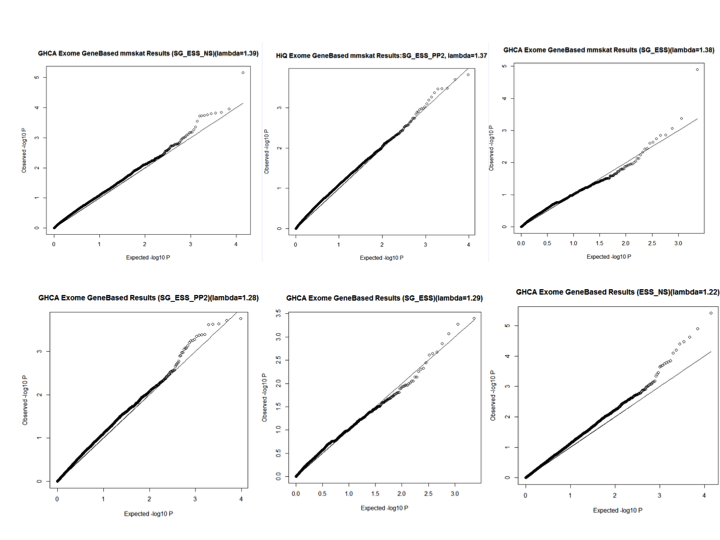


**Supplementary Table 1: Summary of the samples removed at each stage of the quality control process**

| **Quality Control Criteria** | **Number of Samples** | |
| --- | --- | --- |
|  | **Cases** | **Controls** |
| **Original sample number** | 1759 | 6,865 |
| Initial QC (genotyping and GenomeStudio control failures) | 27 | 15 |
| Non-European (by PCA with 1KGP datasets) | 200 | 875 |
| Relatedness (PI-HAT >0.1875) | 37 | 2707 |
| Call Rate (<0.99) | 30 | 0 |
| Intensity Outliers (>4SD from mean) | 53 | 11 |
| Heterozygosity Outliers (>4SD from mean) | 3 | 4 |
| **Total samples remaining** | 1,409 | 3,253 |

**Supplementary Table 2:** Summary of variants removed during the quality control process

| **SNP Quality Control Measures** | **HiQ (exome12v1-1)** | **MTFS (exome12v1)** |
| --- | --- | --- |
| Total Variants | 242,901 | 247,870 |
| Call Rate <0.99 | 188 | 66 |
| Cluster Sep<0.4 | 11,889 | 380 |
| HWE P<1x10^-4^ | 448 | 283 |
| **Additional QC on the merged dataset** | | |
| **Intersecting variants between HiQ and MTFS** | **229,901** | |
| Discordantly monomorphic variants | 4 | |
| Duplicates | 691 | |
| Poor Clustering | 1,083 (533v1 + 550v2) | |
| Insertion/ Deletion mutations | 120 | |
| Tri-allelic variants | 86 | |
| HWE P<1x10^-4^ (repeated after recalling genotypes) | 49 | |
| **Total variants passing QC** | **227,858** | |

Supplementary Table 3: Summary of SNPs included in the analysis and their function. MAC: Minor allele count.

|  | **All SNPs** | **Functional** | **Functional variants included in analyses** | |
| --- | --- | --- | --- | --- |
| **Type** | **Total Variants** | **MAC>0 in HiQ and in MTFS** | **Single SNP (MAF>0.01)** | **Gene-based (MAF<=0.05, MAF>1e-6, MAC>0)** |
| Non-synonymous | 191,571 | 68,270 | 16,992 | 101,246 |
| stop_loss | 222 | 172 | 22 | 95 |
| stop_gain | 4,796 | 1036 | 116 | 2133 |
| start_loss | 457 | 172 | 50 | 256 |
| start_gain | 3 | 2 | 2 | 2 |
| Normal splice site | 144 | 87 | 62 | 54 |
| Essential splice site | 1,846 | 480 | 65 | 858 |
| **Total functional** | **199,039** | **70,112** | **17,309** | **104,644** |
| synonymous | 8,183 | - | - | - |
| Upstream | 11 | - | - | - |
| Downstream | 19 | - | - | - |
| Intron | 5,846 | - | - | - |
| Utr3 | 573 | - | - | - |
| Utr5 | 194 | - | - | - |
| Intergenic | 13,993 | - | - | - |
| **Total Non-Functional** | 28,819 | - | - | - |
| **Total** | **227,858** | **70,112** | **17,309** | **104,644** |

**Supplementary table 4: Publications from which SNPs that had been previously demonstrated to be associated with cognitive related traits were slected. 467 SNPs were identified from 17 publications. Citations marked with * did not report any variants with P values<1x10^-4^, but the studies were listed on GWA catalog and so variants were retrieved from the paper’s supplementary material.**

| **Pubmed ID** | **1^st^ Author** | **Journal** | **No variants** | **Title** |
| --- | --- | --- | --- | --- |
| 23732972* | Hashimoto R | Am J Psychiatry | 0 | Genome-wide association study of cognitive decline in schizophrenia. |
| 23722424 | Rietveld CA | Science | 13 | GWAS of 126,559 individuals identifies genetic variants associated with educational attainment. |
| 23535033 | Sherva R | Alzheimers Dement | 44 | Genome-wide association study of the rate of cognitive decline in Alzheimer's disease. |
| 23358156 | Benyamin B | Mol Psychiatry | 28 | Childhood intelligence is heritable, highly polygenic and associated with FNBP1L. |
| 23207651 | Davies G | Mol Psychiatry | 8 | A genome-wide association study implicates the APOE locus in nonpathological cognitive ageing. |
| 22449649 | Loo SK | J Am Acad Child Adolesc Psychiatry | 10 | Genome-wide association study of intelligence: additive effects of novel brain expressed genes. |
| 22126837 | LeBlanc M | J Psychiatr Res | 2 | Genome-wide study identifies PTPRO and WDR72 and FOXQ1-SUMO1P1 interaction associated with neurocognitive function. |
| 22054870 | De Jager PL | Neurobiol Aging | 1 | A genome-wide scan for common variants affecting the rate of age-related cognitive decline. |
| 21826061* | Davies G | Mol Psychiatry | 251 | Genome-wide association studies establish that human intelligence is highly heritable and polygenic. |
| 21694764* | Martin NW | PLoS One | 49  (+48 genes) | Educational attainment: a genome wide association study in 9538 Australians. |
| 21483430 | Carless MA | Mol Psychiatry | 1 | Impact of DISC1 variation on neuroanatomical and neurocognitive phenotypes. |
| 21107309 | McClay JL | Neuropsycho- pharmacology | 10 | Genome-wide pharmacogenomic study of neurocognition as an indicator of antipsychotic treatment response in schizophrenia. |
| 20306291* | Davis OS | Behav Genet | 9 | A three-stage genome-wide association study of general cognitive ability: hunting the small effects. |
| 20125193 | Cirulli ET | Eur J Hum Genet | 62 | Common genetic variation and performance on standardized cognitive tests. |
| 19734545 | Need AC | Hum Mol Genet | 59 | A genome-wide study of common SNPs and CNVs in cognitive performance in the CANTAB. |
| 18067574* | Butcher LM | Genes Brain Behav | 6 | Genome-wide quantitative trait locus association scan of general cognitive ability using pooled DNA and 500K single nucleotide polymorphism microarrays. |
| 17903297 | Seshadri S | BMC Med Genet | 14 | Genetic correlates of brain aging on MRI and cognitive test measures: a genome-wide association and linkage analysis in the Framingham Study. |

**Supplementary Table 5:** Previously associated variants – only showing SNPs that were represented on the exomechip through either LD (r^2^>0.6) or position within 250kb of the previously reported SNP and P value less than 0.05. EA= Educational Attainment, CP=Cognitive Performance, AD=Alzheimer’s disease,

| **1^st^**  **Author** | **Pubmed**  **ID** | **Trait** | **Chr** | **Pos** | **SNPs** | **Reported**  **p-Value** | **Reported effect** | **LD (r2>0.6) friends to**  **reported SNPs (snp1, pos1, P1,r2)** | **SNPs within 250kb of previous SNP**  **and P<0.05**  **(rsid pos, P, beta, SE)** |
| --- | --- | --- | --- | --- | --- | --- | --- | --- | --- |
| Sherva | 23535033 | AD | 1 | 171557600 | rs2421847 | 9.00E-07 | 0.26 | NA | rs2232816,171753039,0.01955,0.02486,0.01064 |
| Sherva | 23535033 | AD | 7 | 25161602 | rs1861525 | 2.00E-07 | 0.25 | NA | rs877834,25267934,0.01257,-0.02883,0.01155 |
| Sherva | 23535033 | AD | 17 | 45930539 | rs4794202 | 8.00E-08 | 0.19 | NA | rs34480825,45915766,0.008416,0.0808,0.03065 |
| Sherva | 23535033 | AD | 17 | 48692082 | rs117964204 | 1.00E-09 | 0.28 | NA | rs2290862,48557326,0.02249,0.02289,0.01003 |
| Sherva | 23535033 | AD | 22 | 44526105 | rs75617873 | 5.00E-07 | 0.17 | NA | rs2294918,44342116,0.03817,-0.02056,0.009916 |
| Cirulli | 20125193 | CP | 1 | 38247153 | rs12117544 | 8.00E-06 | NR | NA | rs4653328,38227086,0.006048,-0.02768,0.01008 |
| Cirulli | 20125193 | CP | 2 | 231420401 | rs17275498 | 8.00E-06 | NR | NA | rs7590429,231258150,0.007241,0.02428,0.009036 |
| Cirulli | 20125193 | CP | 6 | 30080369 | rs34704616 | 6.00E-06 | NR | rs116129929,30080204,0.2627,1.0 | NA |
| Cirulli | 20125193 | CP | 6 | 121339179 | rs1343075 | 2.00E-06 | NR | NA | rs56300302,121401996,0.03555,0.0826,0.03928 |
| Cirulli | 20125193 | CP | 7 | 116577124 | rs7782376 | 8.00E-06 | NR | NA | rs56391007,116411990,0.00915,-0.0989,0.03793 |
| Cirulli | 20125193 | CP | 9 | 108427062 | rs1463984 | 3.00E-06 | NR | NA | rs41277797,108363426,0.01498,-0.0669,0.02749 |
| Cirulli | 20125193 | CP | 16 | 83757328 | rs3784962 | 3.00E-06 | NR | NA | rs62640905,83999548,0.02654,-0.06171,0.02781 |
| Cirulli | 20125193 | CP | 16 | 83757328 | rs3784962 | 6.00E-06 | NR | NA | rs62640905,83999548,0.02654,-0.06171,0.02781 |
| Cirulli | 20125193 | CP | 20 | 8114704 | rs6118083 | 7.00E-06 | NR | NA | rs2076015,7963041,0.02473,0.04333,0.01929 |
| Need | 19734545 | CP | 1 | 222923351 | rs6683071 | 4.00E-06 | NR | rs2936052,222802376,0.9331,0.718492 | NA |
| Need | 19734545 | CP | 3 | 121344140 | rs3772130 | 6.00E-06 | NR | rs2070180,121351338,0.0598,0.987126 | rs1381057,121154974,0.01394,0.02614,0.01063 |
| Need | 19734545 | CP | 8 | 124831834 | rs10481151 | 4.00E-07 | NR | NA | rs6995099,124710664,0.02111,-0.03922,0.017 |
| Need | 19734545 | CP | 12 | 125033933 | rs12423712 | 7.00E-06 | NR | NA | rs78753849,124798811,0.02053,-0.06563,0.02832 |
| Need | 19734545 | CP | 18 | 40288293 | rs8085804 | 8.00E-06 | NR | NA | rs148544378,40323567,0.009795,-0.09013,0.03488 |
| Need | 19734545 | CP | 21 | 16340289 | rs2229741 | 6.00E-07 | NR | NA | rs139263261,16339852,0.02455,0.08969,0.03988 |
| Seshadri | 17903297 | CP | 1 | 234583618 | rs10489896 | 6.00E-06 | NR | rs4920246,234582651,0.4324,1.0 | NA |
| Seshadri | 17903297 | CP | 17 | 56847945 | rs9303401 | 5.00E-06 | NR | rs17822735,56620167,0.2458,0.642769 | NA |
| Rietveld | 23722424 | EA | 1 | 204576983 | rs11584700 | 8.00E-12 | 1.09 | rs11588857,204587047,0.175,0.978738 | NA |
| Rietveld | 23722424 | EA | 2 | 100818479 | rs4851266 | 5.00E-11 | 1.05 | NA | rs4851287,100915772,0.04167,0.01943,0.009536 |
| Rietveld | 23722424 | EA | 6 | 26510564 | rs1056667 | 2.00E-08 | 0.09 | rs3736781,26505362,0.1931,1.0 | rs4712990,26413744,0.03581,0.03112,0.01482 |
| Rietveld | 23722424 | EA | 6 | 33662295 | rs3227 | 3.00E-07 | 1.04 | **rs4713668,33690796,0.004621,0.650733** | rs4713668,33690796,0.004621,-0.02981,0.01052 |
| Rietveld | 23722424 | EA | 16 | 28837515 | rs8049439 | 1.00E-07 | 0.09 | rs2904880,28944396,0.1318,0.620359 | NA |
| Benyamin | 23358156 | Intelligence | 6 | 32317276 | rs2076540 | 3.10E-05 | NA | rs115521982,32261771,0.8051,0.983595 | NA |
| Benyamin | 23358156 | Intelligence | 17 | 35860991 | rs12946892 | 8.50E-05 | NA | rs12944821,35956391,0.3571,0.532386 | NA |
| Benyamin | 23358156 | Intelligence | 20 | 13696129 | rs6042314 | 7.00E-06 | 0.06 | NA | rs3180370,13747441,0.002883,0.02857,0.009582 |
| Loo | 22449649 | Intelligence | 2 | 127330954 | rs1550404 | 1.00E-06 | 7.05 | NA | rs111631066,127451514,0.03299,-0.06998,0.03281 |
| Loo | 22449649 | Intelligence | 3 | 2895684 | rs11713158 | 9.00E-07 | 5.04 | NA | rs2290610,3139957,0.04497,0.01886,0.009405 |
| Loo | 22449649 | Intelligence | 10 | 64223383 | rs10995170 | 3.00E-07 | 4.42 | NA | rs7076156,64415184,0.03942,0.02134,0.01036 |
| Loo | 22449649 | Intelligence | 22 | 32229649 | rs5994434 | 5.00E-07 | 6.29 | rs140081,32112461,0.6354,0.50956 | rs140079,32110943,0.02957,0.04693,0.02156 |
| Davies | 21826061 | Intelligence | 4 | 57902761 | rs1277311 | 9.04E-07 | 0.1107 | NA | rs61748756,57797350,0.04907,0.06312,0.03206 |
| Davies | 21826061 | Intelligence | 3 | 186184328 | rs6771063 | 3.05E-06 | -0.1052 | NA | rs34522046,186358346,0.02377,-0.08663,0.03831 |
| Davies | 21826061 | Intelligence | 9 | 111435632 | rs11794727 | 9.13E-06 | 0.1905 | NA | rs2230798,111656228,0.01662,0.08555,0.03571 |
| Davies | 21826061 | Intelligence | 16 | 2365288 | rs11867129 | 9.21E-06 | 0.1745 | NA | rs72768728,2547034,0.01358,-0.09989,0.04046 |
| Davies | 21826061 | Intelligence | 2 | 74372678 | rs828888 | 1.08E-05 | -0.0997 | NA | rs17721059,74596527,0.03302,0.07209,0.0338 |
| Davies | 21826061 | Intelligence | 14 | 57826995 | rs11158157 | 2.71E-05 | 0.1006 | NA | rs35759976,57755564,0.03853,0.03689,0.01782 |
| Davies | 21826061 | Intelligence | 2 | 74436721 | rs828903 | 2.89E-05 | 0.0947 | NA | rs17721059,74596527,0.03302,0.07209,0.0338 |
| Davies | 21826061 | Intelligence | 2 | 74374641 | rs828884 | 4.63E-05 | -0.0982 | NA | rs17721059,74596527,0.03302,0.07209,0.0338 |
| Davies | 21826061 | Intelligence | 2 | 74428015 | rs7340453 | 5.74E-05 | -0.0909 | NA | rs17721059,74596527,0.03302,0.07209,0.0338 |
| Davies | 21826061 | Intelligence | 3 | 2925030 | rs17646346 | 6.62E-05 | 0.1531 | NA | rs2290610,3139957,0.04497,0.01886,0.009405 |
| Davies | 21826061 | Intelligence | 9 | 111483336 | rs11791687 | 6.68E-05 | 0.1705 | NA | rs2230798,111656228,0.01662,0.08555,0.03571 |
| Davies | 21826061 | Intelligence | 17 | 76521526 | rs642612 | 7.83E-05 | -0.0888 | NA | rs691652,76491127,0.009503,0.03119,0.01202 |
| Davies | 21826061 | Intelligence | 2 | 74373044 | rs828887 | 7.95E-05 | 0.0942 | NA | rs17721059,74596527,0.03302,0.07209,0.0338 |
| Davies | 21826061 | Intelligence | 4 | 147411428 | rs11737630 | 8.95E-05 | -0.2386 | NA | rs13152799,147560411,0.02074,0.03736,0.01615 |
| Davies | 21826061 | Intelligence | 11 | 1011490 | rs7950955 | 9.1E-05 | 0.088 | NA | rs116256283,1028379,0.02791,0.08893,0.04044 |
| Davies | 21826061 | Intelligence | 19 | 36900778 | rs2271842 | 9.2E-05 | 0.1085 | NA | rs10419469,37134737,0.03968,0.03655,0.01776 |
| Davies | 21826061 | Intelligence | 4 | 62637158 | rs2345049 | 8.64E-06 | -0.0985 | NA | rs35106420,62758491,0.003214,-0.1044,0.03542 |
| Davies | 21826061 | Intelligence | 3 | 149277773 | rs713372 | 1.36E-05 | 0.1094 | NA | rs62622807,149498097,0.02807,-0.08993,0.04094 |
| Davies | 21826061 | Intelligence | 12 | 28095425 | rs11609312 | 1.89E-05 | 0.1061 | NA | rs11049125,27916206,0.03229,0.02954,0.01379 |
| Davies | 21826061 | Intelligence | 12 | 28093217 | rs2347590 | 2.13E-05 | 0.1055 | NA | rs11049125,27916206,0.03229,0.02954,0.01379 |
| Davies | 21826061 | Intelligence | 12 | 28090871 | rs12300399 | 2.22E-05 | 0.1053 | NA | rs11049125,27916206,0.03229,0.02954,0.01379 |
| Davies | 21826061 | Intelligence | 5 | 114451587 | rs2034246 | 2.35E-05 | 0.0909 | NA | rs79290430,114469808,0.004003,0.1184,0.04113 |
| Davies | 21826061 | Intelligence | 4 | 62645032 | rs12509742 | 3.93E-05 | 0.092 | NA | rs35106420,62758491,0.003214,-0.1044,0.03542 |
| Davies | 21826061 | Intelligence | 4 | 62647908 | rs2345043 | 4.85E-05 | 0.0908 | NA | rs35106420,62758491,0.003214,-0.1044,0.03542 |
| Davies | 21826061 | Intelligence | 14 | 92909309 | rs12588868 | 5.38E-05 | 0.0848 | NA | rs45587635,92958522,0.04561,0.0419,0.02096 |
| Davies | 21826061 | Intelligence | 4 | 62638468 | rs2345047 | 6.49E-05 | 0.0893 | NA | rs35106420,62758491,0.003214,-0.1044,0.03542 |
| Davies | 21826061 | Intelligence | 2 | 101023635 | rs4149510 | 7.25E-05 | -0.0838 | NA | rs4851287,100915772,0.04167,0.01943,0.009536 |
| Davies | 21826061 | Intelligence | 12 | 28099396 | rs1861911 | 7.67E-05 | 0.095 | NA | rs11049125,27916206,0.03229,0.02954,0.01379 |
| Davies | 21826061 | Intelligence | 5 | 63877325 | rs7730085 | 9.04E-05 | -0.094 | NA | rs76090587,63665448,0.01038,0.03674,0.01433 |
| Davies | 21826061 | Intelligence | 3 | 149259169 | rs1865604 | 9.45E-05 | -0.0854 | NA | rs62622807,149498097,0.02807,-0.08993,0.04094 |
| Martin | 21694764 | EA | 8 | 17630589 | rs6986402 | 4.43E-05 | unknown | NA | rs209569,17611593,0.02101,-0.1018,0.04409 |

**Supplementary Table 6:** Common SNP (MAF > 0.01) associations with the strongest evidence of association in the discovery analysis (P_CC_ < 1x10^-3^).

| **SNP** | **MARKER_ID** | **MAF** | **P VALUE** | **BETA** | **SEBETA** | **CTRLCNT** | **CASECNT** |
| --- | --- | --- | --- | --- | --- | --- | --- |
| rs28379706 | 22:50728062_T/C_Nonsynonymous:PLXNB2 | 0.400 | 1.31E-05 | -0.041 | 0.009 | 1104/1572/577 | 597/619/193 |
| rs921135 | 11:57798723_A/G_Nonsynonymous:OR6Q1 | 0.299 | 9.65E-05 | -0.043 | 0.011 | 1550/1369/334 | 765/540/104 |
| rs2513726 | 11:57798942_A/G_Nonsynonymous:OR6Q1 | 0.299 | 9.65E-05 | -0.043 | 0.011 | 1550/1369/334 | 765/540/104 |
| rs1042391 | 6:16290761_T/A_Nonsynonymous:GMPR | 0.413 | 1.20E-04 | -0.036 | 0.009 | 1070/1589/594 | 525/692/192 |
| rs12574273 | 11:12315256_A/C_Nonsynonymous:MICALCL | 0.020 | 1.83E-04 | 0.118 | 0.032 | 3149/102/2 | 1326/80/2 |
| rs3735478 | 7:44800176_G/T_Nonsynonymous:ZMIZ2 | 0.282 | 3.50E-04 | 0.036 | 0.010 | 1719/1287/247 | 702/568/139 |
| rs117111102 | 10:96447920_C/T_Nonsynonymous:CYP2C18 | 0.013 | 3.67E-04 | 0.141 | 0.039 | 3191/62/0 | 1350/57/2 |
| rs2485652 | 1:44595410_A/G_Nonsynonymous:KLF17 | 0.374 | 4.79E-04 | 0.034 | 0.010 | 1322/1497/434 | 515/665/229 |
| rs2230625 | 1:12186058_A/G_Nonsynonymous:TNFRSF8 | 0.020 | 4.92E-04 | 0.112 | 0.032 | 3148/103/2 | 1334/74/1 |
| rs17723637 | 9:109687403_A/G_Nonsynonymous:ZNF462 | 0.140 | 5.14E-04 | -0.048 | 0.014 | 2358/824/71 | 1092/298/19 |
| rs138377917 | 8:143763531_G/A_Stop_Gain:PSCA | 0.038 | 5.92E-04 | 0.082 | 0.024 | 3032/218/3 | 1280/127/2 |
| rs10024123 | 4:71232430_T/C_Nonsynonymous:SMR3A | 0.044 | 6.93E-04 | -0.075 | 0.022 | 6/248/2999 | 7/140/1262 |
| rs3814541 | 9:109689752_C/T_Nonsynonymous:ZNF462 | 0.139 | 8.27E-04 | -0.046 | 0.014 | 2363/820/70 | 1092/298/19 |

**Supplementary Table 7:** Analysis results for rs28379706, located in *PLXNB2*. The C allele is associated with higher intelligence. The units for β coefficients are IQ points. The effect size is given in standard deviation units. Variance explained is the trait variance based on the effect size.

| **Discovery** |  | |  | | | | | | | | | | | | | | | | | | |
| --- | --- | --- | --- | --- | --- | --- | --- | --- | --- | --- | --- | --- | --- | --- | --- | --- | --- | --- | --- | --- | --- |
| **Analysis** | | **Cases** | **Controls** | **Sample Size** | | **Minor/Major allele** | | | **MAF Cases** | **MAF Controls** | | **OR**  **(95% CI)** | | |  | | **Variance Explained** | |  | **P** | |
| Case/Control EMMAX | | HiQ | MTFS | 1409/3253 | | C/T | | | 0.356 | 0.419 | | 0.76  (0.69-0.83) | | |  | | 0.0016 | |  | 1.76E-05 | |
| **Extension** |  | |  | | | | | | | | | | | | | | | | | | |
| **Analysis** | | **Study** |  | **Sample Size** | | **Minor/Major allele** | | | **MAF** |  | | **β**  **(+/- s.e.)** | | | **Effect Size (+/- s.e.)** | | **Variance Explained** | | **SE** | **P** | |
| QT  Linear regression | | MTFS |  | 3253 | | C/T | | | 0.419 |  | | -0.816  (0.358) | | | -0.057 (0.025) | | 0.0014 | | 0.00026 | 0.0267 | |
|  |  | TEDS |  | 4072 | | C/T | | | 0.398 |  | | -0.198  (0.3378) | | | -0.013 (0.023) | | 6.940E-05 | | 0.00020 | 0.5574 | |
|  |  | ALSPAC |  | 6461 | | C/T | | | 0.398 |  | | -0.02  (0.29) | | | -0.0013  (0.018) | | 6.726E-07 | | 0.00013 | 0.944 | |
| **Combined** |  | |  | |  | |  |  | | |  | |  |  | |  | |  | | |  |
| **SNP** | | **Study** |  |  | | **Minor/Major**  **allele** | | | **MAF** |  | | **β**  **(+/- SE)** | | | **Effect Size**  **(+/-SE)** | | **Variance Explained** | | **SE** | **P** | |
| QT  Meta-analysis | | MTFS/TEDS  /ALSPAC |  |  | | C/T | | | 0.403 |  | | -0.2932  (0.1875) | | | -0.0183  (0.0123) | | 1.349E-04 | | 0.00006 | 0.1365 | |

**Supplementary Table 8:** Gene centric analysis of rare variants. Genes with the strongest evidence of association (P_GCC_ < 5 x 10^-5^) in the discovery study.

| **Gene** | **Location** | **Variant Class** | **Test** | **p-value** |
| --- | --- | --- | --- | --- |
| ENPP2 | 8:120575249-120638927 | StopGain_ESS_NS | CMC | 3.90E-06 |
| EHD2 | 19:48220194-48244447 | StopGain_ESS_NS | SKAT-O | 6.82E-06 |
| SERPINA10 | 14:94754643-94756669 | StopGain_ESS | SKAT-O | 1.25E-05 |
| IL1R2 | 2:102625068-102644771 | StopGain_ESS_NS | CMC | 1.27E-05 |
| EHD2 | 19:48220194-48244447 | StopGain_ESS_NS | CMC | 2.37E-05 |
| ADAMTS13 | 9:136287582-136323080 | StopGain_ESS_NS | CMC | 3.40E-05 |
| ARFGAP3 | 22:43195114-43227602 | StopGain_ESS_NS | CMC | 3.99E-05 |
| ZNF276 | 16:89789073-89806477 | StopGain_ESS_NS | CMC | 6.31E-05 |
| CASC1 | 12:25261702-25347959 | StopGain_ESS_NS | CMC | 8.04E-05 |
| KCNQ1 | 11:2549229-2869129 | StopGain_ESS_NS | SKAT-O | 0.00010983 |
| MAN2A2 | 15:91448591-91463000 | StopGain_ESS_NS | CMC | 0.0001436 |
| DNAH8 | 6:38702283-38998040 | StopGain_ESS_NS | SKAT-O | 0.00014411 |
| SNX19 | 11:130750592-130785593 | StopGain_ESS_PP2Damaging | SKAT-O | 0.00015122 |
| SERPINA10 | 14:94754684-94756912 | StopGain_ESS_NS | SKAT-O | 0.00015218 |
| COMP | 19:18893732-18900759 | StopGain_ESS_NS | CMC | 0.0001563 |
| ENPP2 | 8:120575249-120638927 | StopGain_ESS_NS | SKAT-O | 0.00015934 |
| UQCR10 | 22:30163410-30163571 | StopGain_ESS_NS | CMC | 0.0001672 |
| UQCR10 | 22:30163410-30163571 | StopGain_ESS_NS | SKAT-O | 0.0001723 |
| SNX19 | 11:130750592-130785593 | StopGain_ESS_PP2Damaging | CMC | 0.0001777 |
| BCL2L10 | 15:52404650-52404706 | StopGain_ESS_NS | CMC | 0.0001803 |
| BCL2L10 | 15:52404650-52404706 | StopGain_ESS_NS | SKAT-O | 0.00018294 |
| IL1R2 | 2:102625068-102644771 | StopGain_ESS_NS | SKAT-O | 0.00018568 |
| COMP | 19:18893732-18900759 | StopGain_ESS_NS | SKAT-O | 0.00019315 |
| SKOR1 | 15:68118338-68119427 | StopGain_ESS_PP2Damaging | CMC | 0.0001949 |
| SKOR1 | 15:68118338-68119427 | StopGain_ESS_PP2Damaging | SKAT-O | 0.00019823 |
| ZNF880 | 19:52877677-52888282 | StopGain_ESS_NS | CMC | 0.0001994 |
| KCNQ1 | 11:2549229-2869129 | StopGain_ESS_NS | CMC | 0.0002125 |
| BIRC7 | 20:61867669-61870553 | StopGain_ESS_NS | CMC | 0.0002279 |
| CALR | 19:13050026-13051246 | StopGain_ESS_PP2Damaging | CMC | 0.0002331 |
| ARHGAP22 | 10:49654462-49687709 | StopGain_ESS_PP2Damaging | CMC | 0.0002391 |
| CCDC11 | 18:47753755-47788535 | StopGain_ESS_PP2Damaging | CMC | 0.000245 |
| PRSS23 | 11:86518714-86663341 | StopGain_ESS_NS | SKAT-O | 0.00028355 |
| FAM126A | 7:22985242-23018030 | StopGain_ESS_PP2Damaging | SKAT-O | 0.00032912 |
| PRKCSH | 19:11547212-11559428 | StopGain_ESS_PP2Damaging | SKAT-O | 0.00033724 |
| DGKH | 13:42701612-42795514 | StopGain_ESS_PP2Damaging | SKAT-O | 0.00034165 |
| SERPINA10 | 14:94754684-94756912 | StopGain_ESS_NS | CMC | 0.0003541 |
| THNSL2 | 2:88472791-88485632 | StopGain_ESS_NS | CMC | 0.0003959 |
| GUCA1C | 3:108672558-108672558 | StopGain_ESS | CMC | 0.0004012 |
| SLC25A47 | 14:100792507-100795978 | StopGain_ESS_PP2Damaging | CMC | 0.0004118 |
| C8orf82 | 8:145753161-145753161 | StopGain_ESS_PP2Damaging | CMC | 0.0004146 |
| MMP8 | 11:102584135-102595487 | StopGain_ESS | SKAT-O | 0.00041949 |
| C8orf82 | 8:145753161-145753161 | StopGain_ESS_PP2Damaging | SKAT-O | 0.00042016 |
| KCNH4 | 17:40312063-40330841 | StopGain_ESS_PP2Damaging | CMC | 0.0004216 |
| GDF11 | 12:56137479-56142553 | StopGain_ESS_NS | SKAT-O | 0.00043746 |
| FRS3 | 6:41738889-41740658 | StopGain_ESS_PP2Damaging | CMC | 0.0004557 |
| DNAH8 | 6:38702283-38998040 | StopGain_ESS_NS | CMC | 0.0004593 |
